# The impact of functional imaging on radiation medicine

**DOI:** 10.1186/1748-717X-3-25

**Published:** 2008-09-15

**Authors:** Nidhi Sharma, Donald Neumann, Roger Macklis

**Affiliations:** 1 Research fellow, Department of Radiation Oncology, Cleveland Clinic Foundation, 9500 Euclid Avenue, Cleveland, OH 44195, USA; 2 Staff physician, Department of Nuclear Medicine, Cleveland Clinic Foundation, 9500 Euclid Avenue, Cleveland, OH 44195, USA; 3 Professor of Medicine (Radiation Oncology), Cleveland Clinic Lerner College of Medicine and Department of Radiation Oncology, 9500 Euclid Avenue, Cleveland, OH 44195, USA

## Abstract

Radiation medicine has previously utilized planning methods based primarily on anatomic and volumetric imaging technologies such as CT (Computerized Tomography), ultrasound, and MRI (Magnetic Resonance Imaging). In recent years, it has become apparent that a new dimension of non-invasive imaging studies may hold great promise for expanding the utility and effectiveness of the treatment planning process. Functional imaging such as PET (Positron Emission Tomography) studies and other nuclear medicine based assays are beginning to occupy a larger place in the oncology imaging world. Unlike the previously mentioned anatomic imaging methodologies, functional imaging allows differentiation between metabolically dead and dying cells and those which are actively metabolizing. The ability of functional imaging to reproducibly select viable and active cell populations in a non-invasive manner is now undergoing validation for many types of tumor cells. Many histologic subtypes appear amenable to this approach, with impressive sensitivity and selectivity reported.

For clinical radiation medicine, the ability to differentiate between different levels and types of metabolic activity allows the possibility of risk based focal treatments in which the radiation doses and fields are more tightly connected to the perceived risk of recurrence or progression at each location.

This review will summarize many of the basic principles involved in the field of functional PET imaging for radiation oncology planning and describe some of the major relevant published data behind this expanding trend.

## Review

### Introduction and background

Recent advances in high precision radiation treatment methodologies have focused on developing a tighter correspondence between the visualized location of neoplastic target structures and the radiation dose deposition patterns chosen in an attempt to control the target tissue proliferation. The ability to map the real time or near-real-time positional information has been facilitated by the rapid growth over the last few decades in high speed computing and algorithms for shape recognition and manipulation. These processing algorithms are gleaned from diverse fields including industrial manufacturing, military applications, and the entertainment industry. These advances have now essentially made it possible to "paint" recognizable target structures with modulated pulses of ionizing radiation using the complex beam-shaping routines developed for intensity modulated radiotherapy (IMRT). The validity of such dose painting is, however, currently the source of intense debate. In order to determine the optimal dose deposition patterns, methods are required to correlate three dimensional anatomic structures with function, physiology, and change over time. The use of PET (positron emission tomography) provides one important medical methodology being optimized for this purpose. This review will summarize the current status of the incorporation of physiologic "functional" medical imaging into radiation medicine and radiotherapy treatment plan design.

Though PET is not really a new field, it has recently undergone a dramatic revitalization as new clinical indicators are validated for this type of functional imaging. The principles behind PET involve the non-invasive analysis and positional correlation of biochemical processes, typically with a level of quantization not easily achieved using other nuclear medicine methodologies. This superiority is based on the fact that PET uses the positron-emitting annihilation event that occurs when an electron and positron collide and vanish with the creation of two opposed photons of a precise characteristic energy 511 keV. This sort of annihilation reaction can be demonstrated in natural radioisotopes such oxygen-15, fluorine-18, and carbon-11. The invention of complex detectors capable of sensing the emitted energy stream allowed PET to be validated as a reproducible physiologic biomarker, originally for cardiac and neuroanatomic studies and more recently for many physiologic processes found in oncology. The high sensitivity of PET for cancer processes relates to the partially planned and partially fortuitous discovery that the glucose analog fluorodeoxyglucose (FDG) accumulates in most human cancers and is physiologically "trapped" within the cell by phosphorylation. Positron radio-labeled ^18^FDG provides some of the highest signal-to-noise ratios observed in the sometimes murky domain of oncology imaging due to factors such as neoplastic over-expression of glucose transport proteins, increased glycolysis (the "Warburg Effect") and modified cellular hexokinase activity. The kinetics of this trapping process produces a gradual rise in the signal and the resolution limit of the image (typically several millimeters) produces an imaging envelope representing the total region in which abnormal glycolysis patterns may be differentiated from baseline metabolism. There is a delayed physiologic signal (typically becoming maximal after several hours or more) and reasonable quantitation may be achieved by calculating the "standardized uptake value" (SUV) which normalizes signal size to infused isotope dose and patient mass. While the typical PET signal produced by FDG uptake cannot be considered specific for neoplasia, the PET process has the tremendous advantage over other oncologic imaging methods of producing rapid whole-body images capable of delineating and differentiating between normal structures and many different sites of primary cancers and metastatic disease. Though the half-lives of PET radiopharmaceuticals are typically very short (< 0.5 hr) the test may be repeated in a serial fashion in order to define a valid time course for the observed physiologic processes. Thus, for the investigator interested in signature cancer biomarkers, PET provides an entirely new dimension of physiologic information that may be highly complementary to the routine 3-D anatomic information obtained through volume-based methods such as CT, ultrasound, and MRI. Table 1 shows some of the primary Medicare-accepted indications for the use of this test. For the radiation oncologist, functional information such as ^18^FDG-PET thus provides much useful data on oncologic process in addition to tumor location. PET has been used as an adjunct to traditional anatomic modalities to more accurately assess local and regional disease extent and to detect early sites of metastasis. Preoperative evaluation of regional metastases has been tested in a number of disease sites, including the axilla [1,2] in breast cancer, the neck in squamous cell carcinomas of head and neck, [3,4] and the liver in colorectal carcinoma [5,6]. FDG-PET has been most extensively studied in non-small cell lung cancer (NSCLC), where surgical assessment of the mediastinal lymph nodes is typically performed before definite resection. Using appropriately designed and informative reporter molecules, PET can be used to trace the evolution of the sorts of abnormal physiologic signals which are often considered the metabolic hallmark of the transformation event.

**Table 1:** Medicare-accepted indications (2007) for positron emission tomography (PET) for Cancers

**INDICATION**	**PURPOSE**
Breast cancer	Staging, restaging, evaluating treatment response
Colorectal cancer	Diagnosis, staging, restaging
Esophageal cancer	Diagnosis, staging, restaging
Head and neck cancer	Diagnosis, staging, restaging
Lung cancer	Diagnosis, staging, restaging
Lymphoma	Diagnosis, staging, restaging
Melanoma	Diagnosis, staging, restaging
Solitary pulmonary nodules	Characterization
Thyroid cancer	Restaging(with negative iodine-131 scan and positive thyroglobulin)

### Basis of PET scan technology

With the push for new of technology in the fields of nuclear medicine and radiation oncology, the PET scan has become a valuable modality in the hands of the physicians. It has proved to be of immense importance in modifying the radiation treatment therapy for patients with malignancies. The basic principle of oncologic PET scan is based on the characteristic of the malignant cells which may divide continuously in an uncontrollable manner, thus altering their metabolic profile compared to the normal cells. In the past, numerous radiological tracers have been put to practice, but presently 2-[18 F]-fluoro-2-deoxy-D-glucose (FDG) is the most popular one. Its role in functional imaging is unique, as it helps differentiate groups of active cancer cells, allowing further imaging and intervention in the specific diseased site. Across oncological applications, the sensitivity and specificity of FDG-PET ranged from 84 to 87% and 88 to 93% respectively [7].

**Figure 1:**
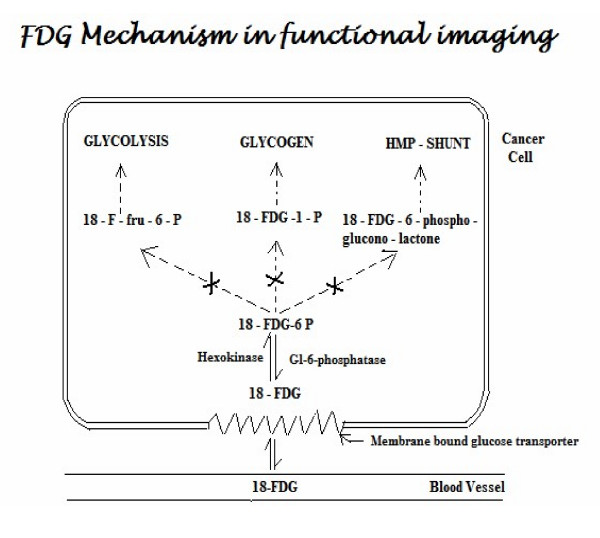
**FDG Mechanism in Functional Imaging**. **Abbreviations**: 18-FDG: 2-[18 F]-fluoro-2-deoxy-D-glucose; Gl: Glucose; Fru: fructose.

Upon its intravenous administration, the membrane bound glucose transporter takes up FDG into the cells, where it gets phosphorylated to ^18^FDG-6-phosphate by the enzyme hexokinase. This product cannot enter the glycolytic pathway and thus keeps accumulating inside the cells (See Figure 1). The uncontrolled proliferation and metabolic activity of the tumor cells is picked up by PET scan as it detects the photons emitted by radiotracers like ^18^FDG (or C-11, N-13 etc.). These photons are emitted at a specific energy (511 keV) in opposite directions. Therefore, PET scanners have detectors placed on the opposite sides of the region from where the photons are emitted (within the patient) and the detectors register an event only if both the detectors record the photon emission at the same time [8].

There are a few limitations of the PET-only images like lack of anatomic details required for therapy, physiologic update of FDG by normal tissues, fat, muscle and lymphoid tissue, increasing confounding and also lack of an easy method to incorporate this information into treatment planning.

### Roles for PET imaging in radiotherapy

#### Malignant lymphoma

The role of PET and PET-CT in oncology is currently most fully embodied in the relevant work on malignant Hodgkin's and non-Hodgkin's Lymphoma. For Hodgkin's Lymphoma staging, ^18^FDG-PET was shown to be somewhat more useful than other more traditional anatomic imaging technologies such as CT and MRI and has been claimed recently to be the "most accurate imaging technology for staging malignant lymphoma." It is now fairly routine to obtain a pretreatment baseline ^18^FDG-PET study for Hodgkin's and aggressive non-Hodgkin's Lymphoma prior to the initiation of chemotherapy and ^18^FDG-PET studies have largely replaced gallium scans as a pretreatment and post-treatment whole-body radionuclide studies for lymphoma. While some of the earliest studies evaluating ^18^FDG-PET for malignant lymphoma date from the 1980s, investigation in this area has expanded dramatically in the last decade as evidence mounted for the sensitivity and cost effectiveness of the technology. For malignant lymphoma, both tumor grade and proliferative activity appeared to be somewhat correlated with the uptake intensity of the FDG signal. However, these findings have not always been reproducible and at present it appears that the correlation of high SUV levels to tumor grade are still insufficient to be used in clinical treatment decision making.

In addition to providing a sensitive and noninvasive tool for oncologic staging, FDG-PET has also shown utility in assessing response to treatment. This is particularly helpful in-lymphoma, where post-treatment fibrosis can obscure detection of residual disease [9,10]. In a study of 44 patients with abdominal presentations of Hodgkin's disease (HD) and non-Hodgkin's lymphoma (NHL) [11], FDG-PET proved superior to anatomic imaging in determining post-treatment tumor viability. Thirty seven of the 44 patients had residual CT abnormality following chemotherapy with or without radiation therapy. Thirteen patients were also shown to be positive by FDG-PET, and all of these patients eventually relapsed. Only 1 patient, negative by FDG-PET but positive by CT, relapsed. The relapse-free survival rate was 0% for those patients positive by FDG-PET, and 95% for those negative by FDG-PET at 2 years. Clearly, patients shown to have residual disease by FDG-PET should be considered for additional treatment.

The role of FDG-PET in Hodgkin's Lymphoma workups and management has been the subject of several recent reviews. Castellucci et al evaluated 967 consecutive PET studies in 706 individual patients treated previously for malignant lymphoma. They found that over 20 percent showed focal FDG uptake unrelated to the presence of known tumor deposits (e.g., a "false positive"). This "false positive" uptake appeared to result from a number of potential causes including either "brown fat" (mean SUV: 11.7) thymic hyperplasia (mean SUV: 4.1) muscle contraction (mean SUV: 7.4) or various types of inflammation or infection (mean SUV levels 4–7) [12]. These authors suggest that the use of correlated single-platform PET-CT should minimize the number of spurious "false positives" produced by non-tumor FDG signals. At a minimum, it suggests that FDG hot-spots should not be evaluated in the absence of additional anatomic information.

FDG-PET can also serve as a sensitive means to monitor therapy in progress, with an eye to changing ineffectual treatments in midcourse. A provocative study from Germany used early response to FDG-PET to predict outcome. The treatment course of 11 patients with NHL was monitored by Romer et al [13]. All patients underwent FDG-PET imaging before treatment, at 1 week, and again at 6 weeks. The mean decrease in SUV at day 42 was 79%. Interestingly, the tumor SUV levels at week 1 were significantly lower in the group of 6 patients remaining in remission after 16 months follow-up, than in the group of patients eventually relapsing. Patients showing no response by FDG-PET at 1 week might be candidates for more aggressive/altered treatment regimens. Others have used FDG-PET in a similar fashion to monitor response to neoadjuvant chemotherapy in patients with locally advanced breast cancer [14,15].

**Figure 2:**
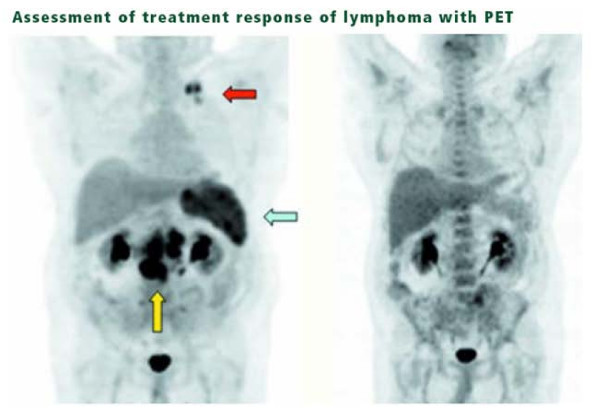
**Assessment of treatment response of lymphoma with PET**. Images of pre- and post-therapy PET scans in a lymphoma patient treated with chemotherapy. The pre therapy image (left) shows increased FDG uptake in the left supraclavicular region (red arrow), mesentery, retroperitoneum (yellow arrow), and spleen (olive arrow). The post-therapy image (right) shows no residual disease, with a bone marrow activation commonly seen after chemotherapy and which can be seen with other treatments such as granulocyte colony-stimulating factor.

For evaluation of response, the PET or PET-CT appears to be gaining ground with respect to accepted clinical utility. The "International Workshop Criteria for Response in NHL" recently adopted PET as the "gold standard in response evaluation." For NHL patients treated with CHOPR chemotherapy, response after just 2–3 cycles was shown to predict eventual clinical outcomes. This "early look" at response is of extreme importance in choosing therapies likely to produce long-term control without the necessity of a protracted and potentially dangerous course of treatment. Other investigators are evaluating F-18 fluorothymidine (^18^FLT) rather than ^18^FDG due to the more specific uptake of this analog into DNA [16]. While FDG mirrors glycolysis, ^18^FLT is thought to mirror DNA synthesis. Patients with positive PET studies after chemotherapy had a significantly higher risk of relapse than those with negative scans (P < 0.0001) though not all patients with persistently positive scans ultimately showed evidence of clinical progression and a negative post-treatment PET was not an accurate predictor that local progression was contained (See Figure 2).

For radiotherapy, one interesting question is whether the PET studies can be used to pick out those patients who might benefit from post-chemotherapy involved-field radiation, and whether the location and intensity of the PET signal can be used to guide radiotherapy treatment planning. Kahn et al [17] showed that FDG-PET was useful in identifying the patients likely to recur and the sites at which they were most at risk for recurrence. However, patients with positive post-chemotherapy PET studies were not fully protected by local field radiotherapy as administered in this trial. The authors note that the fields were designed to include only the persistent PET-positive regions of assumed disease, and that dose and fractionation schemes were "highly individualized" with median doses of 30.6 Gy and dose ranges of 9–46 Gy. Over half of the relapses observed in this study occurred infield. Thus, either the treated region or the dose was insufficient to control disease sites showing post-chemotherapy positive PET signals.

With respect to radiotherapy field design, some have discussed the use of PET and other similar functional studies in what they call "Theragnostic imaging" appropriate for use as a guide for radiation "dose painting." The term Theragnostic is meant to refer to the use of medical images to guide treatment decisions and intensity. For radiotherapy, the suggestion is that tumor burden and clonogen density may be indicated by FDG or FLT PET SUV required levels and that these levels may be used as a proxy for recurrence risk and therefore required dose of radiation necessary to achieve local control [18]. If this conjecture proves true, the deliberately inhomogeneous dose delivery algorithms currently used in IMRT technology may be fitted using "inverse planning" to estimated risk maps incorporating indices of proliferation, hypoxia, and other known local recurrence risk factors. In a sense, this is a more dosimetrically rigorous version of the now-accepted risk-adjustment methodology commonly used in current clinical radiotherapy approaches in which NHL complete responders (CR) to chemotherapy are given lower doses than patients showing only partial responses. Whether this general principle, clinically validated for aggressive lymphomas, can be applied to small sub-portions of non localized tumors will require additional study. One could construct reasonable arguments to support the hypothesis that either the FDG-intense areas or the FDG-cold areas would require higher doses, depending on whether one proposes to dose-intensify regions of higher proliferation or lower oxygenation. While specific PET markers of hypoxia such as ^18f^Misonidazole are currently being studied in both pre-clinical and clinical trials, some investigators claim that images obtained on untreated patients may show significant changes over a few hours or days ("intermittent hypoxia") and hence are not reproducible markers of a fixed biology [19]. If the hypoxia markers show us only temporary biologic indications of intermittent vascular status then dose adjustments based on these images would be invalid. The idea of dose-painting based on "theragnostic imaging" though intellectually appealing, is thus still in the hypothesis stage and will require substantial clinical validation before it can be incorporated into clinical practice. Several recent sets of authoritative guidelines have now appeared emphasizing the importance of PET imaging in the interpretation of lymphoma responses [20-22].

### Specific tumor types

#### Head and neck tumors

FDG-PET has an expanding role in head and neck cancer management as it provides improved staging, treatment response delineation and recurrence detection for a wide range of solid cancers [23] including head and neck disease [24]. It has excellent sensitivity and specificity rates (96% and 98.5%) for cervical nodal staging [25]. In comparison to FDG-PET, the sensitivity and specificity of CT and MRI were lower in many studies, ranging from 64% [26] to 95% [27] and from 41% [28] to 97% [27], respectively. Post treatment FDG-PET is often of great value in predicting residual viable tumor [29]. Early work from a number of groups suggests that FDG-PET/CT disease targeting can help assist conformal radiotherapy and IMRT planning in several diseases including head and neck disease [30]. Lowe et al. investigated 44 patients with head-and-neck tumors after primary radio chemotherapy. A year after treatment, FDG-PET showed viable tumor tissue in 16 cases and histological data confirmed the diagnosis made by PET. The sensitivity was 100% for FDG-PET and 38% for CT plus MRI. The specificity of FDG-PET was 93% and of CT and MRI 85% [31]. Kunkel et al. found a significant correlation between FDG uptake after neoadjuvant radiation treatment and histological response of mouth carcinoma [32]. Also, Nishioka et al. showed that the integration of FDG-PET in radiation treatment planning for oropharyngeal (twelve patients) and nasopharyngeal (nine patients) carcinomas may also cause a reduction in the radiation fields. The GTV for primary tumor was not changed by image fusion in 19/21 patients (90%). Of the nine patients with nasopharyngeal cancer, the GTV was enlarged by 49% in only one patient and decreased by 45% in one patient. In 15/21 patients (71%) the tumor-free FDG-PET detection allowed normal tissue to be spared. Particularly, parotid glands were spared and, thus, xerostomia could be avoided. The authors concluded that the image fusion between FDG-PET and MRI/CT was useful for encompassing the whole tumor area in the irradiation field and for sparing of normal tissue in GTV, CTV and PTV determination [33]. FDG-PET/CT provides more accurate assessment than CT imaging of treatment response and in high index suspicion patients, PET-CT performed within four weeks after radiotherapy treatment were highly predictive for residual disease [34]. FDG-PET can also aid in determining response to organ preservation treatment in head and neck cancer, where true disease status after radiation is often obscured by fibrosis. Greven et a1 [35] reviewed the utility of FDG-PET in 31 patients suspected of persistent disease after definitive radiation therapy for carcinoma of the larynx. The overall sensitivity of FDG-PET was 80% and the specificity was 81%. The authors concluded that potentially morbid post-treatment biopsy can be postponed in FDG-PET-negative patients, despite clinical evidence of persistent disease. Similarly, Farber et a1 [36] reviewed their experience with 28 patients with head and neck cancers treated with definitive radiation therapy, all suspected of harboring recurrent/persistent disease. Twelve of 13 patients with FDG-positive scans had biopsy-proven active disease; 2 of 15 patients with negative PET imaging did have residual disease, yielding an overall accuracy of 89%. Others have also observed high sensitivity and specificity values for FDG-PET in a similar setting of suspected residual/recurrent disease after definitive treatment [37,38]. Thus the results of FDG-PET imaging can guide early intervention following treatment, potentially at a stage when surgical salvage is still possible.

#### Breast tumors

Breast cancer is the most common cause of cancer death in women in the western world and imaging is essential for its diagnosis and staging. Also, most of the patients need adjuvant chemo-radiation therapy as a standard of care. The increasing experience with PET scanning in breast cancer patients is revealing a significant role for this imaging modality. PET plays an important role in investigation of metastatic disease and evaluation of pathological response to various chemotherapeutic regimens. According to Wolfort et al, for patients with stages II and III breast cancer who present with a suspicion for recurrent disease, a whole-body FDG-PET scan may act as a useful adjunct in the evaluation of recurrence. However, its added benefit over conventional imaging can be questioned [39]. PET has proved superior to conventional imaging modalities and has a high positive predictive value for the axillary lymph nodes involvement, especially patients with advanced tumors [40,41]. According to Port *et al*, conventional imaging and PET were equally sensitive in detecting metastatic disease in patients with high-risk, operable breast cancer, but PET generated fewer false-positive results [42]. In this pilot study GCPET has been shown to be feasible in a district general hospital, enabling the provision of a limited on-site PET imaging service. In the cases studied it was more sensitive than ultrasonography or mammography. GCPET may provide additional information that could be important in planning the management of some patients with breast cancer [43]. According to a study conducted by Kawada et al, there is increase in the metabolic activity of the tumors in patients who experienced clinical benefits on treatment with lapatinib. Thus, FDG-PET may be useful for the evaluation of molecular targeted drugs, such as lapatinib [44]. Also, in patients with breast cancer and rising tumor markers, FDG-PET/CT was superior to CT and had high performance indices for diagnosis of tumor recurrence [45].

For the radiation oncologist, one important message provided by this new information relates to decisions concerning the need to include various nodal groups (e.g. internal mammary chains) within primary treatment fields. Several investigators are now evaluating this question in a systematic fashion [46].

#### Lung tumors

**Figure 3:**
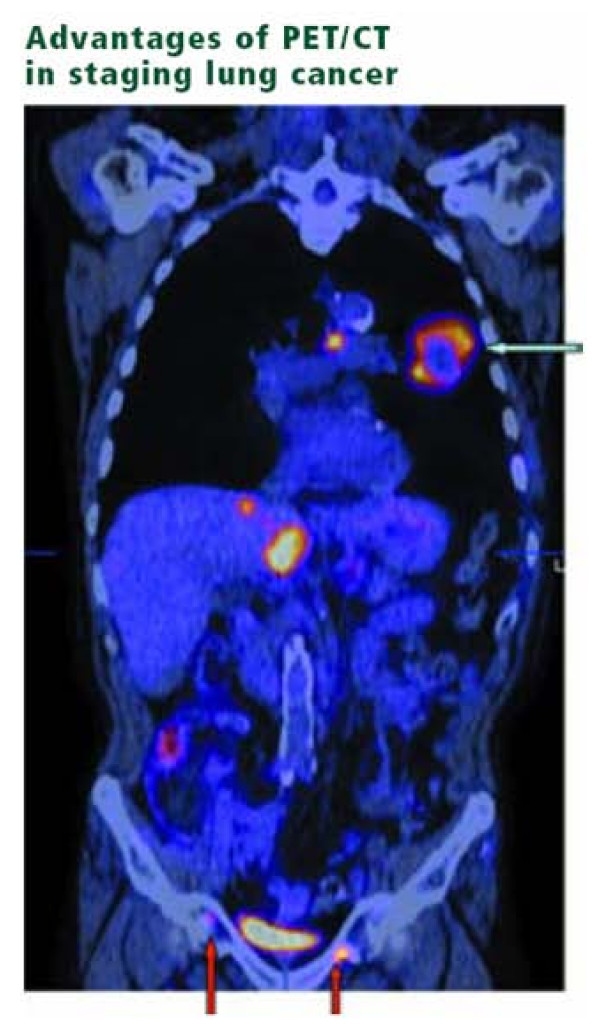
**Advantages of PET/CT in staging Lung cancer**. Coronal slice of a PET/CT scan demonstrating a large left lung mass showing peripheral hypermetabolism with central necrosis (olive arrow), positive mediastinal disease, two liver lesions, and previously unsuspected pelvic bone metastases (red arrows). The presence of distant metastases changes the treatment options for the patient.

Lung cancer is the major cause of deaths in United States with patients presenting at an advanced stage. PET presents a dramatic advance in imaging of lung cancers. PET has an excellent negative predictive value of 87–100% for Non-small cell lung cancer. Recently, Weber et al. reviewed all clinical trials published between 1995 and 2002 for the use of FDG-PET for preoperative staging of patients with non-small cell lung cancer (NSCLC) according to the criteria of evidence-based medicine. The value of FDG-PET in the diagnosis of lymph node metastases in patients with NSCLC was investigated in 16 studies including 1,355 patients and corresponded to the criteria of the Agency for Health Care Policy and Research. The mean sensitivity and specificity of FDG-PET were 85% (81–89%) and 87% (83–91%), respectively. In the studies comparing FDG-PET and CT, the mean sensitivity and specificity of CT alone remained at 66% (58–73%) and 71% (65–76%), respectively. Compared to "conventional" CT-based staging, the results of FDG-PET correctly modified the tumor stage in 17% of the patients. The tumor stage was incorrectly diagnosed by FDG-PET in only 2% of the patients [47]. Additionally, the PLUS multi centric randomized trial showed that the addition of PET to conventional work-up prevented unnecessary surgery in 20% patients with suspected NSCLC [48]. PET scan improves the detection of distant metastasis over conventional staging [49]. Additionally, FDG-PET plays an effective role in predicting accurate response to chemo radiation and neoadjuvant therapy and assessing aggressiveness of the tumor, thereby defining treatment options [50]. Also, PET sets the gold standard in evaluation of an indeterminate solitary pulmonary nodule or mass where PET has proven to be significantly more accurate than CT to distinguish between benign and malignant lesions [51]. It also improves pre-operative staging of respectable lung metastasis (See Figure 3). In Small cell Lung cancer, the role of PET is not completely established. According to Hauber et al [52], PET was equivalent to the battery of staging procedures done conventionally. Craig et al [53] reported that patients were actually down staged based on PET results. PET-CT plays a vital role in identifying mesothelioma patients who respond to treatment improved over CT alone [54]. Ten studies pointed out the significant implications of FDG-PET in staging lymph node involvement.

FDG-PET is also useful in the noninvasive evaluation of distant metastatic disease in lung cancer. Erasmus et al, at Duke University [55], studied 27 patients with known SCLC and an adrenal mass shown on conventional imaging (mean size, 3 cm). FDG-PET identified metastatic disease in 25 of 33 lesions, "23 of which were confirmed positive by biopsy. All lesions negative by PET were also negative histologically (sensitivity, 100%). In a cohort of 94 patients at the University Hospital, Zurich, prospectively evaluated by FDG-PET imaging for mediastinal involvement, 4~14% were found to have distant metastatic disease that was not shown by conventional CT.

These findings are supported by data in the literature, showing an advantage of FDG-PET in lung cancer staging over CT [56]. PET is thus a promising imaging modality for patients with extensive disease and poor prognoses, making treatment more efficacious.

#### Gastro-intestinal tumors

The advent of PET imaging has also led to significant advances in staging of GI malignancies. FDG-PET plays a vital role in detecting metastatic disease in esophageal cancer with overall accuracy of 82% and high specificity and sensitivity levels exceeding other conventional staging modalities [57].

It has maximum benefit for patients with locally advanced disease in whom a curative surgery can treat the patient. It also has great potential in predicting histopathological response to neo-adjuvant therapy and in monitoring the radiofrequency ablation success soon after intervention [49].

In gastric cancer, FDG-PET helps in detecting distant metastasis such as to liver, lung, adrenals, ovaries and skeleton [58].

**Figure 4:**
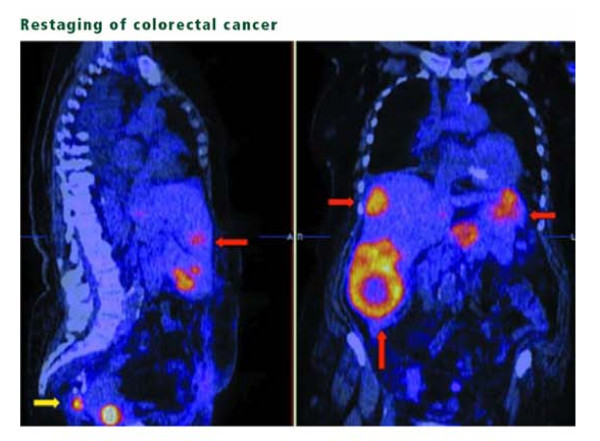
**Restaging of colorectal cancer**. Sagittal (left) and coronal (right) PET/CT slices of patient with prior surgery and increasing carcinoembryonic antigen show increased FDG uptake in multiple liver lesions(red arrows), as well as recurrence of local disease in the presacral space (yellow arrow).

With advent in research, 18F-FDG-PET detects metastases in colorectal cancer patients and helps decide a better treatment plan to prolong their survival. Early 18F FDG-PET can predict pathological response to pre-operative treatment [40] (See Figure 4). Also, automated segmentation of PET signal from rectal cancer may allow immediate and sufficiently accurate definition of a preliminary working planning target volume(PTV) for pre-op radiotherapy [41].

PET has not proved of much assistance in diagnosis of pancreatic malignancy but it can help in detection of metastases [59]. FDG-PET helps identify two distinct scintigraphic patterns of focal and uniform uptake that predict the presence of diffuse or nodular Peritoneal Carcinomatosis [60].

#### Brain tumors

A main challenge in the management of brain tumors lies in the localization of the extent of tumor and assessment of the functional status of the surrounding brain. Carbon-11-labeled methionine (MET), iodine-123-labeled α-methyl-tyrosine (IMT) and fluorine-18-labeled O-(2) fluoroethyl-L-tyrosine (FET) are the most important amino acids playing a major role in detection of Gliomas. C-11 Methionine PET improves the target volume delineation of meningiomas treated with stereotatic fractionated radiotherapy [24]. Also, the use of PET and PET-CT in conjunction with functional MRI has greatly aided in the management of different brain tumors. Herholz et al. showed a sensitivity and specificity of MET-PET in differentiating between non tumoral tissue and low-grade gliomas of 76% and 87%, respectively [61]. FDG PET is of limited use in brain tumors as the uptake of FDG by normal brain tissue is high, making it indistinguishable from the tumor tissue. But still, DiChiro et al [62] and Alavi et al [63] showed that the amount of FDG uptake in the tumor tissue correlates to the histological grading of the tumor and has prognostic implications. FDG-PET has been evaluated in the planning of radiation with Intensity modulated radio-surgery and radiotherapy with Simultaneous Energy Boost (SEB). FET-PET reliably distinguishes between post therapy benign lesions and tumor recurrence after initial treatment of low- and high-grade gliomas [64]. For meningiomas, which usually occur in the tentorium, orbit, sella, falx cerebri, there is a problem in defining the tumor extension as the normal tissue in these areas gives the same contrast enhancement as the tumor tissue. Recently, it was demonstrated that by using MET-PET/CT fused images, meningioma borders can be more accurately defined in correlation to critical normal organs [65,66].

#### Gynecological tumors

Gynecological malignancies often present a challenge due to their late presentations and insidious nature of symptoms. PET has been shown to be superior to CT alone in staging of cervical cancer [67]. Whole-body FDG PET is a sensitive and specific tool for the detection of recurrent cervical cancer in patients who have clinical findings suspicious for recurrence [68]. Reinhardt et al. found a positive predictive value (PPV) for nodal involvement of 90% with FDG-PET compared to 64% with MRI in non treated patients with cervical cancer [69]. More recently, Dehdashti et al. were the first to demonstrate in 14 cervical cancer patients, an NPV of enhanced Cu-ATSM uptake for the response to treatment [70]. FDG PET was also found to be superior to CT in the evaluation of pelvic and para-aortic lymph nodes. CT-PET guided IMRT has been used to develop treatment plans to deliver radiotherapy to positive para-aortic region lymph nodes [71]. In Gestational trophoblastic neoplasia, FDG-PET is potentially useful for providing precise metastatic mapping of tumor extent, monitoring response and localizing viable tumors after chemotherapy [72]. In ovarian cancer, Bristow et al [73], evaluated uses of PET in detecting clinically occult but surgically resectable disease. They found that its ability to localize persistent disease and failure to identify small volume disease was useful in selecting patients who are candidates for cytoreductive surgery. In vulvar cancer, a prospective PET study evaluating the detection of groin metastases has been reported [74]. PET-CT thus may alter the management of patients with a variety of gynecologic malignancies.

#### Renal and urological tumors

Currently FDG-PET has a limited role in diagnosis of prostate cancer mainly because of the low uptake of FDG in the tumor and normal excretion of FDG through urine. Visualization of prostate cancer with current imaging methods (CT, MRI, and ultrasonography) is severely impaired [75]. The low glucose uptake, the significant overlap of tracer uptake in tumor and in the benign prostate hyperplasia, and the renal excretion of FDG into the bladder limit the diagnosis of prostate cancer using FDG-PET [76-78]. FDG-PET appears to be promising in the assessment of lymph nodes and bone metastases [79]. Morris et al. showed that FDG-PET can differentiate osseous metastases from scintigraphically quiescent lesions [80]. However, the results of FDG-PET in early stages of prostate cancer are not satisfactory for tumor detection, and other tracers have been intensively evaluated in the recent past. The development of new tracers and technical improvements will probably make PET imaging a viable diagnostic tool in prostate cancer and renal cell carcinoma [81]. C-11 acetate and C-11 choline seem to be the two promising tracers playing an important role in Prostate cancer. In patients with primary testicular cancer, PET can be used in conjunction with conventional imaging techniques to diagnose retroperitoneal masses. FDG-PET has shown very encouraging results in a limited number of studies, and has also demonstrated a good sensitivity for initial staging. FDG-PET seems to be superior to conventional imaging modalities for detecting local disease and recurrence, and distant metastases [79].

### Incorporation of functional information into the radiation medicine treatment planning

Though formal radiation therapy treatment planning techniques date from the earliest days of the 20^th ^century, the current era of reliable dosimetry and treatment planning can conveniently be bookmarked beginning with the rise of the mini-computer and micro-computer and the associated software developed in the 1970s. Rather than the rough dosimetric approximations and look-up tables previously used for "ballpark" dosimetric analyses, we are now in an era of physical rigor going far beyond the initial impressionistic estimates. As 3-D target localization experience grew, the original question of target volume projection into a series of planar two-dimensional spaces was replaced by a much more sophisticated hierarchy of deliberately planned target volumes including the "surgical" or "gross" target volume (GTV), the "pathologic" or "expanded clinical" target volume (CTV) and the real-world "corrected" or "planning" target volume (PTV). Each of these enlarging tissue volumes represented a finer-tuned understanding of what one must do to make the radiation dose deposition matrix correspond with the known and expected clonogenically viable tumor regions. These target sub-volumes included the dosimetric impact of various poorly visualized "microscopic disease" regions (included within the CTV) and dosimetric uncertainties due to expected target movement and radiation edge effects (seen within the PTV).

The addition of the PET information allows a new, more realistic target volume to be defined based on a kind of probability envelope indicating the tissue region undergoing the metabolic processes defining the "biologic target volume" (BTV). This "BTV" indicates the region in which the described physiology is readily demonstrable. In oncology, the most common "BTV" represents the area of abnormal glucose metabolism indicated by FDG-PET and related processes. While very non-specific, many different kinds of neoplasms have now been shown to display markedly abnormal glucose metabolism and the sensitivity and specificity detectible in the non-invasive imaging of this process is on the order of 80 to 90 percent for many tumors. Surprisingly, this sensitivity and specificity may rival or exceed that of CT or MRI for certain physiologically active tumors such as lung cancer. The recent popularization of dual-platform PET-CT detectors now allows sub-centimeter correlation between the source of the PET signal and the anatomic region responsible for that signal [82].

In designing appropriate radiotherapy target volumes, it is apparent that the extra cost and difficulty of utilizing the BTV to define the treatment volume will only be justified if the clinical data show that the application of the BTV approach will add information that is actually new (versus simply redundant with anatomic imaging techniques). This appears to be the case. The Agency for Healthcare Policy and Research (AHPR) investigated 16 studies incorporating information on over 1,000 patients and compared staging data from PET or PET-CT to data obtained using CT information alone for lung cancer patients. In 17 percent of cases, the FDG-PET correctly modified tumor stage. The use of this methodology to cancel or modify potentially toxic surgical approaches in tumors which later displayed occult metastatic spread was reduced by over fifty percent. Multiple cost effectiveness analyses based on this sort of data have concluded that the incremental costs associated with the use of PET-CT were justifiable and in accord with other well-accepted principles used for medical economics [8,9]. For diagnostically difficult cases with CT-indicated enlargement of regional lymph nodes, the use of functional imaging would be especially useful if it proved reliable. However, the relative lack of PET specificity in patients with other known causes for physiologic inflammation makes this method too unreliable to depend on. At present, it appears that PET-based target volume definition is fraught with difficulty in any circumstance with active inflammation. This unfortunately includes many postoperative settings and situations with benign causes of immune system activation.

## Conclusion

The field of radiation medicine and nuclear imaging are both progressing rapidly with respect to technologic sophistication and multi-platform interface capabilities. Radiation oncology has previously incorporated multiple imaging methodologies including: CT, ultrasound, and MRI into the treatment planning process to allow highly accurate and serially updated beam-direction instructions. This field is now known as "image-guided radiation therapy" (IGRT) and can be seen as further evolutionary progression in the quest to maximize dose delivered to true target tissue and minimize the dose delivered to nearby non-target tissues. A near-term future goal is now the incorporation of functional imaging methods such as 18 FDG-PET in the same fashion. Multiple recent studies are appearing in literature attesting to the value of incorporating PET-CT information in radiotherapy treatment planning [83-87]. This will allow a determination of the degree of physiologic activity located within various sub-components of presumed target tissue. As more and more types of tumor targets are validated for this sort of functional and predictive analysis, functional imaging is likely to enter the main stream as a critical tool for radiation medicine field design and as an accepted non-invasive surrogate endpoint appropriate for clinical trial design and clinical decision-making. All of these advances can be seen as way-stations on the road to the effective, non-invasive, minimally toxic, and ultimately personalized cancer medicine.

## Competing interests

The authors declare that they have no competing interests.

## Authors' contributions

RM and NS have contributed to conception and design, acquisition, analysis and interpretation of data. RM, DN and NS have been involved in drafting the manuscript and revising it critically for important intellectual content. RM and NS have given final approval of the version to be published.
